# Construction Methods of Mesoscopic Models for Concrete and Quantitative Analysis of Mesoscopic Damage

**DOI:** 10.3390/ma19071392

**Published:** 2026-03-31

**Authors:** Xiaoli Wang, Shutao Li, Yeqing Chen, Shang Ma, Jialin Chen

**Affiliations:** 1 State Key Laboratory of Target Vulnerability Assessment, Beijing 100036, China; wangxiaoli18@163.com (X.W.); sma_1002@hrbeu.edu.cn (S.M.); cjl0321@yeah.net (J.C.); 2 Institute of Defense Engineering, Academy of Military Sciences (AMS), PLA, Beijing 100036, China

**Keywords:** concrete, mesoscopic model, interfacial transition zone (ITZ), damage evolution, quantitative analysis

## Abstract

**Highlights:**

- A 2D mesoscopic concrete model considering randomly distributed aggregate grains has been established.
- A quantitative relationship between damage energy and the decay of strength and stiffness has been proposed.
- The effects of aggregate geometry, spatial distribution, and ITZ on mechanical properties have been studied.

**Abstract:**

Existing mesoscopic numerical models still exhibit shortcomings in terms of the aggregate geometric fidelity, interface transition zone (ITZ) characterization, and modeling efficiency. To solve these problems, this paper establishes a two-dimensional mesoscopic model and analysis method for concrete, considering randomly distributed convex polygons of aggregate grains and a three-phase structure comprising aggregate, mortar, and ITZ. An efficient random placement algorithm based on background meshing is proposed to enable rapid and accurate model construction. The effects of aggregate geometry, spatial distribution, and ITZ on mechanical properties and damage evolution have been systematically studied. A quantitative relationship has been established between damage energy and the decay of strength and stiffness, and damage quantification indices have been proposed. The damage rates of mortar and ITZ, along with the variation characteristics of the damage variable *d*_c_ at each stage, have been quantified. Neglecting the ITZ leads to overestimation of the peak strength and stiffness of concrete while exacerbating its post-peak brittle behavior. The most significant increases occur in both stiffness decay and damage growth at 90% of peak stress. A sudden change occurs at approximately 0.17% axial strain (corresponding to 80% of peak stress). This study offers a meso-scale foundation for understanding concrete failure and designing high-performance concrete.

## 1. Introduction

Concrete, as the most widely utilized composite material in modern civil engineering, exhibits macro-scale mechanical behaviors and failure modes that are predominantly governed by the nonlinear interactions among its meso-scale constituents of mortar, coarse aggregate, and the interfacial transition zone (ITZ) [1,2,3]. As the weakest phase in the three-phase system, the microstructure and mechanical properties of ITZ play a decisive role in determining the strength, deformation, and damage fracture processes of concrete [4,5]. Owing to the high heterogeneity and multi-scale characteristics inherent in concrete’s internal structure, its failure mechanism is extremely complex, constituting a major research focus in solid mechanics [6,7,8].

Conventional macroscopic homogeneous constitutive models struggle to accurately characterize the local stress concentration and damage evolution within concrete induced by the random distribution of aggregate grains and interfacial effects [9]. In recent years, numerical methods based on meso-mechanics have offered effective approaches for revealing the damage and failure mechanisms of concrete [10,11]. By explicitly characterizing individual components and interfaces, these methods can directly simulate the initiation, propagation, and coalescence of microcracks [12,13,14]. However, existing meso-scale models still exhibit limitations in geometric realism, rationality of interface characterization, and depth of mechanistic interpretation. Most studies simplify aggregate grains as circular or elliptical particles [15,16], which deviates from the actual polygonal morphology. This may underestimate stress concentration at edges, affecting the accurate prediction of damage paths. Moreover, many investigations neglect the interfacial transition zone or oversimplify its mechanical parameters [17,18,19,20], failing to adequately reveal the critical influence of this weak phase on the macroscopic nonlinear response of concrete, particularly in terms of post-peak softening and energy dissipation. Zhou and Hao [21,22] systematically investigated the tensile, compressive, and explosive problems of the mesoscopic model of concrete under different loading conditions. They pointed out that the bonding interface layer between aggregate and mortar would first exhibit failure cracks during compression, and it had the greatest impact on the failure mechanism and tensile strength of concrete during tension. Finally, traditional methods face challenges in aggregate intrusion detection and interface mesh generation, making it difficult to balance model accuracy and computational efficiency [23].

While three-dimensional meso-scale models offer a more realistic simulation of the spatial failure patterns of concrete and their stress–strain responses that correspond more closely with experimental results, they present challenges such as complex modeling procedures, high computational costs, and difficulties in visualizing damage evolution processes [24,25,26]. The comparison between the two-dimensional model and the three-dimensional model is as follows. (a) In aggregate morphology processing, the mainstream method of 2D modeling is to simplify the modeling of circles/ellipses. The 3D model can realize the modeling of the real aggregate morphology (based on CT scan and 3D point cloud reconstruction). (b) ITZ modeling method: the mainstream of 2D modeling is explicit thin-layer modeling, which represents the ITZ by dividing the aggregate edge into fixed-thickness meshes. The 3D model can realize high-precision explicit modeling, which represents the ITZ by 3D thin-layer meshes or zero-thickness interface units. (c) Energy dissipation mechanism: 2D models mainly consider macroscopic energy response (such as total strain energy and total dissipation energy). 3D models can accurately analyze the plastic dissipation, damage dissipation, and interface dissipation caused by crack spatial propagation, and the energy forms are more comprehensive. (d) Quantitative damage analysis: 2D models mainly characterize planar damage and often use the damage variable *d*_c_ as an indicator. 3D models can realize quantitative damage analysis in 3D space and often use indicators such as the 3D damage tensor and spatial damage rate.

The 2D model can clearly and intuitively display the microscopic paths of crack initiation, propagation, and penetration. It is simple to model and has high computational efficiency. It is suitable for conducting systematic analysis of microscopic parameters and large-sample computational studies [22]. The 2D model cannot simulate the damage and failure modes of concrete under complex three-dimensional stress states, and it is difficult to reflect the interlocking and slip effects of aggregate grains and the crack spatial propagation characteristics of concrete in 3D space. The aggregate volume ratio and gradation design of the 2D model are converted from the 3D model, which differs from the actual 3D microstructure of concrete. This study employs a two-dimensional meso-scale modeling approach, focusing on the damage evolution mechanisms and their quantitative characterization in concrete.

In response to the aforementioned research gaps, this study aims to establish a numerical model capable of accurately reflecting the three-phase mesostructure of concrete and the realistic morphology of aggregate grains. It systematically investigates the influence mechanisms of mesostructural characteristics on the macro-mechanical properties and damage evolution processes of concrete. First, a random convex polygonal aggregate grain generation algorithm is established to replicate the true aggregate geometric morphology. The background mesh method is employed to optimize the aggregate placement process, enabling efficient and precise modeling of the three-phase medium-aggregate, mortar, and ITZ. Second, combining experimental data with parameter inversion methods, the mechanical parameters of meso-scale components have been calibrated to validate the reliability of the model. Building on this foundation, the study systematically examines the effects of aggregate morphology and random distribution characteristics on the macro-mechanical behavior of concrete. It reveals both the common patterns and specific mechanisms underlying meso-scale damage evolution. Additionally, the intrinsic relationship between damage energy evolution and the degradation of strength and stiffness is clarified. The damage rates of mortar and ITZ, along with the variation characteristics of the damage variable *d*_c_ at each stage, have been quantified. Furthermore, it quantitatively investigates the impact of ITZ mechanical properties on overall damage behavior.

The methodological innovation is as follows. A high-efficiency algorithm for random convex polygonal aggregate placement based on a background grid is proposed, and a framework for quantitative analysis of concrete mesoscopic damage is established. The innovative mechanism reveals the regulatory mechanism of aggregate shape on the microscopic damage mode and energy dissipation of concrete, clarifies the dominant role of ITZ in the microscopic damage evolution of concrete, and discovers the three-stage law and key characteristic points of the microscopic damage evolution of concrete. By developing a numerical model that accurately reflects the true mesostructure of materials, this study not only provides clear numerical evidence and mechanistic explanations for a deeper understanding of the physical essence of concrete failure but also establishes a parametric modeling and analysis framework that offers theoretical foundations and methodological support for performance-based design and optimization of concrete materials.

## 2. Concrete Mesoscopic Model Construction and Parameter Calibration

### 2.1. Generation of Random Convex Polygonal Aggregate Grains

**Figure 1:**
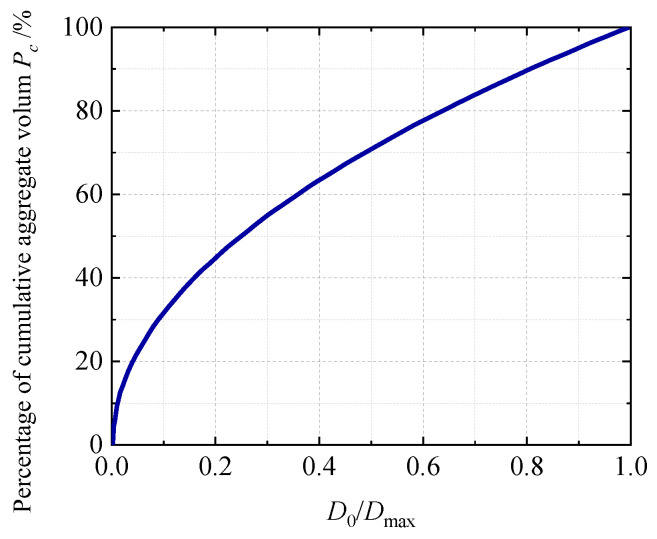
Fuller gradation curve.

The particle size and gradation characteristics of aggregate grains have been characterized by a gradation curve, which takes the aggregate particle size as the variable and reflects the relative content of aggregate grains not exceeding a certain size. In the early 20th century, the American scientist, Fuller, proposed the Fuller curve of maximum density gradation, as shown in Figure 1. This curve is suitable for three-dimensional aggregate gradation design, enabling concrete to achieve an optimal balance between density and strength [27]. Due to the high computational cost associated with three-dimensional meso-scale numerical concrete models, it is common practice to transform the problem into two-dimensional planar models using probabilistic and statistical methods. The core assumption for transforming the Fuller curve into a two-dimensional area distribution is that the aggregate in concrete is a randomly and uniformly distributed convex body, and its projection onto the two-dimensional cross-section is a random convex polygon. There is a definite statistical correlation between the three-dimensional volume proportion of the aggregate and the area proportion of the two-dimensional cross-section, and this correlation is not significantly affected by the specific shape of the aggregate [2,28]. The potential error of this conversion mainly stems from the randomness of the spatial distribution of the three-dimensional aggregate and the projection effect of the two-dimensional cross-section. Walraven and Reinhardt [29] established a correlation model between the volume fraction of aggregates in concrete specimens and the area fraction of aggregate in cross-sections, achieving the transformation of the Fuller gradation curve into the probability distribution of aggregate particle sizes in two-dimensional cross-sections.

(1) $$P_{c}\left(D<D_{0}\right)=P_{k}\left(1.065{\left(\frac{D_{0}}{D_{\max}}\right)}^{0.5}-0.053{\left(\frac{D_{0}}{D_{\max}}\right)}^{4}-0.012{\left(\frac{D_{0}}{D_{\max}}\right)}^{6}-0.0045{\left(\frac{D_{0}}{D_{\max}}\right)}^{8}+0.0025{\left(\frac{D_{0}}{D_{\max}}\right)}^{10}\right)\;$$

where *P*_k_ denotes the volume fraction of aggregate in the total volume of concrete. *D*_0_ denotes the particle size of graded aggregate grains. *P*_c_ represents the occurrence probability of different aggregate gradations on the two-dimensional cross-sections in the experiment. In this study, Equation (1) is adopted to allocate the particle sizes of aggregate grains.

**Figure 2:**
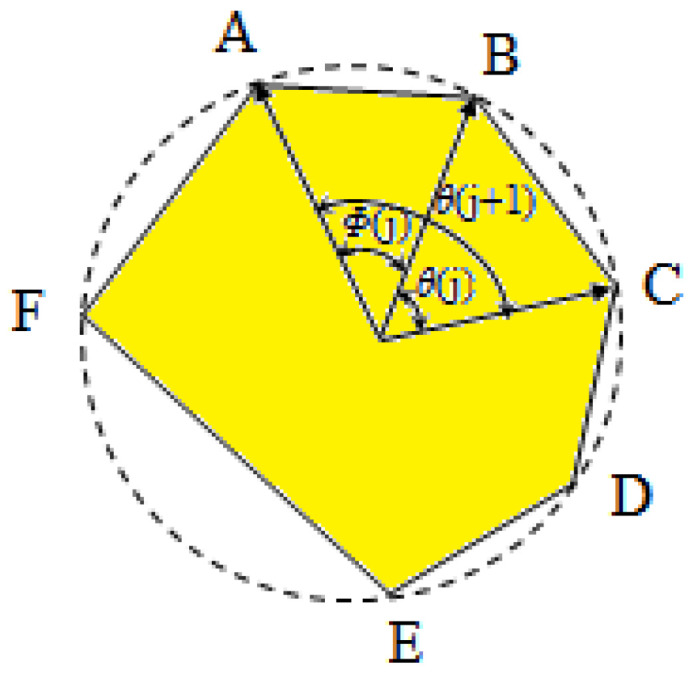
Schematic diagram of a convex polygonal aggregate grain generated by inscribing in a circle.

The dimension of the concrete specimens employed in this study is 150 mm × 150 mm. Following the principle that the maximum aggregate size in a two-dimensional planar model should not exceed 1/3 of the specimen dimension, the aggregate particle size range is determined to be 10 mm to 30 mm. To enhance the efficiency of random aggregate placement, increase the aggregate content, and reduce the frequency of intrusion judgment, a sequential placement strategy from larger to smaller sizes is adopted, ensuring that the aggregates’ gradation satisfies the requirements specified in Equation (1). Furthermore, a novel random generation method for convex polygonal aggregate grains is proposed in this study. Several vertices are randomly selected on the circular boundary and connected in sequence to form a convex polygon inscribed within the circle. The position of each vertex is defined by its polar angle *θ*(*j*) [30], as illustrated in Figure 2.

The included angle *Ф*(*j*) corresponding to any side of the convex polygon is defined as *Ф*(*j*) = *θ*(*j* + 1) − *θ*(*j*). Given that the convex polygon has n edges, the average value of *Ф*(*j*) is 2*π*/*n*. Assuming that *Ф*(*j*) varies around this average value, it can be expressed as:

(2) $$Ф\left(j\right)\;=\frac{2\pi }{n}+\left(2\mathrm{rand}-1\right)\delta \cdot \frac{2\pi }{n}$$

To satisfy the condition that the sum of all *Ф*(*j*) equals 2π, the following correction is applied to *Ф*(*j*):

(3) $$\bar{Ф}\left(j\right)=Ф\left(j\right)\cdot \frac{2\pi }{\sum_{i=1}^{n}Ф\left(i\right)}$$

After the correction, the polar angle of each vertex is given by:

(4) $$\theta \left(j\right)=a+\sum_{i=1}^{j-1}\bar{Ф}\left(i\right)$$

where rand denotes a uniformly distributed random number in the interval [0, 1]. The fluctuation coefficient *δ* is the fluctuation amplitude coefficient of the polar angle of the convex polygonal particle vertex, characterizing the degree of deviation of the central angle *Ф*(j) corresponding to each side of the aggregate from the average value 2*π*/*n*. The value ranges from 0 to 1. An elevated *δ* induces a more pronounced angularity and increases the heterogeneity of edge lengths, thereby escalating the overall geometric irregularity. The choice of *δ* = 0.5 is informed by the geometric statistics of real-world crushed aggregate grains. At this level, the generated interior angles and edge distributions closely match those of engineering-grade aggregate. Referring to existing research on concrete microstructure modeling [30,31], the reasonable range for *δ* is 0.4~0.6. This study uses the intermediate value of 0.5, balancing geometric realism and modeling universality. *a* represents the orientation phase angle of aggregate grains, following a uniform distribution over [0, 2*π*]. *j* is the vertex number of the convex polygonal aggregate. The vertex coordinates of each convex polygonal aggregate are extracted, and the distance between each pair of vertices is calculated. The maximum distance is taken as the characteristic size of the aggregate. Aggregate grains are sequentially counted and tallied compared against preset gradation ranges to determine the quantities of aggregate within each gradation interval.

### 2.2. Random Aggregate Placement Method Based on Background Meshes

**Figure 3:**
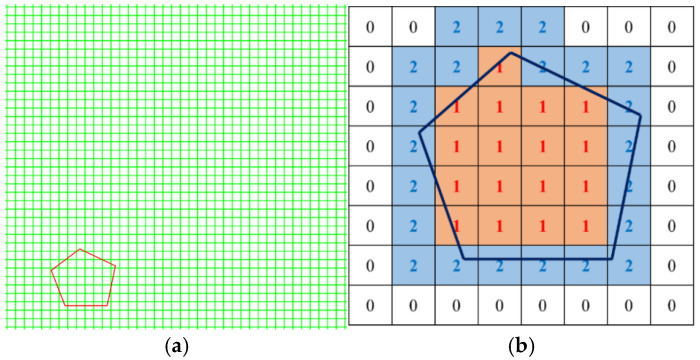
Background mesh and aggregate placement rules. (**a**) Background mesh; (**b**) property judgment for aggregate placement.

**Figure 4:**
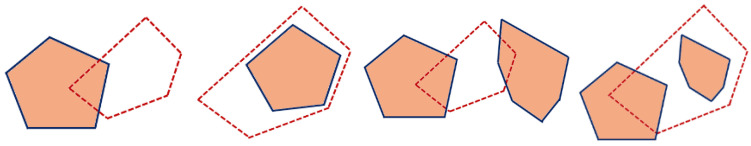
Aggregate particle intrusion mode.

**Figure 5:**
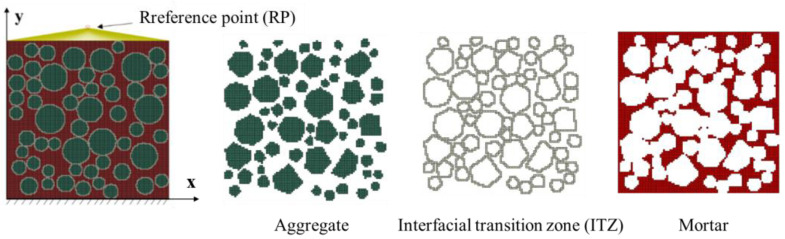
Uniaxial loading on a mesoscopic model of concrete with a three-phase medium.

**Figure 6:**
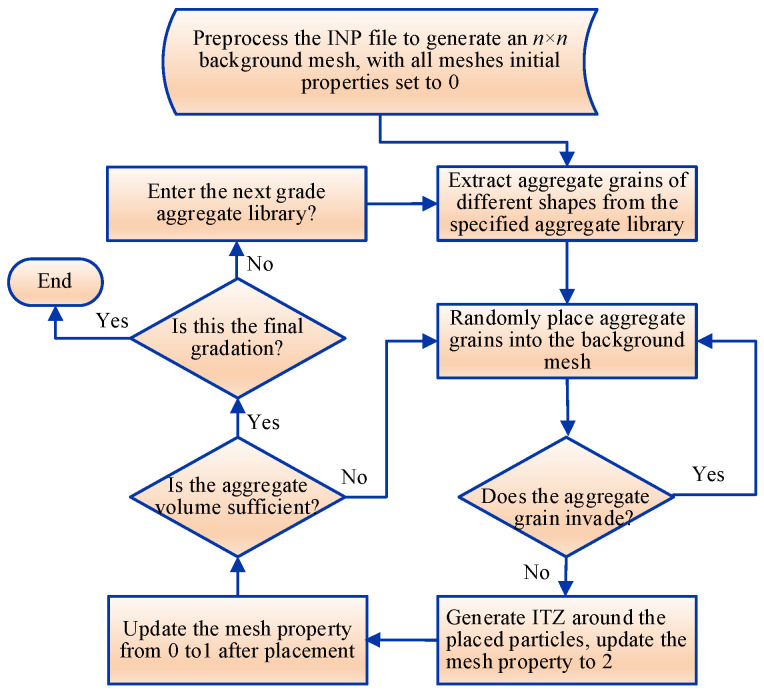
Flowchart of the generation algorithm for the mesoscopic model of concrete’s three-phase medium.

In this study, a random aggregate placement method based on the background mesh is proposed to construct the meso-scale concrete model. A program is written in Python version 3.9 to automatically generate a two-dimensional meso-scale model for the three-phase medium: aggregate, mortar, and ITZ. The specific construction approach is as follows:

1. Preprocessing the INP files in ABAQUS version 2022: A two-dimensional square placement area with a side length of L is generated, which is divided into an *n* × *n* background mesh. The numbers and coordinates of nodes/elements are stored simultaneously. The size of the background mesh is taken as 1/5 to 1/10 of the minimum aggregate diameter [28]. Combined with the minimum aggregate particle size of 10 mm adopted in this study, the mesh size is determined to be 1–2 mm.
2. Editing the inp file with Python: Initialize the element information list, including element number, node number, coordinate, area, and mesh properties. The mesh property values corresponding to mortar, aggregate, and ITZ are denoted by 0, 1, and 2, respectively. In the initial state, the property value of all meshes is set to 0 (no aggregate).
3. Aggregate placement rules: Randomly select a mesh with an initial property value of 0 as the center of the aggregate grain, ensuring that all polygonal vertices of the aggregate are within the square area and that no aggregate grains are placed on the outermost mesh layer. The core reason is to avoid direct contact between aggregate grains and the loading boundary, which could lead to unrealistic stress concentration and disrupt the uniform stress transfer across the loading surface. The constraint is assumed to eliminate the artificial mechanical effects of the loaded boundary. Upon successful placement of an aggregate grain, the property value of the mesh is updated to 1. For the edge meshes of the aggregate, if the area proportion of the aggregate exceeds 50%, the property value is updated to 1. Otherwise, it is updated to 2 (ITZ). The full assignment range of mesh property values is illustrated in Figure 3.
4. Intrusion judgment of aggregate grains: After the placement of the first aggregate grain, subsequent placements require intrusion judgment. Four potential intrusion forms between new and existing aggregate grains are illustrated in Figure 4. The orange particles represent the already placed aggregate grains, while the red dotted-line particles denote aggregate grains awaiting placement. The classification of aggregate intrusion is divided into two steps: Step 1: Mesh attribute pre-screening. First, based on the spatial positional relationship between the mesh and the aggregate (such as whether the mesh center point is within the aggregate outline and the distance between the mesh boundary and the aggregate outline), potential intrusive meshes are quickly screened out, and meshes without intersection are excluded, which greatly improves the judgment efficiency. Step 2: Precise verification by geometric Boolean operation. For the potential intrusive meshes screened in Step 1, a geometric Boolean operation is used to calculate the actual intersection of the mesh unit with the aggregate outline and the thin ITZ region on a mesh-by-mesh basis. Only when the intersection area/mesh area is greater than half of the mesh area is it judged as a valid intrusive mesh, avoiding errors caused by misjudgment due to mesh attributes. The placement and positioning are carried out in the order of larger gradation aggregate grains first, and then smaller gradation aggregate grains. When the placement area of aggregate of each gradation reaches the preset value, the aggregate feeding is terminated.
5. Generation of ITZ: Relevant scholars have confirmed that the thickness of ITZ cannot be ignored [32]. The role of the ITZ in mesoscopic simulation is to characterize the mechanically weak interface between aggregate and mortar rather than to accurately reproduce its geometric thickness. Sensitivity analysis [4,5] verified that the ITZ thickness range of 0.5–1 mm has no significant impact on the macroscopic mechanical properties and microscopic damage evolution of concrete, meeting the accuracy requirements of this study. In the numerical simulation, an equivalent thickness of 1 mm is used. This value is a common setting for microscale simulation of concrete [2,28]. This avoids the problems of excessively fine mesh and drastically increased computational costs associated with modeling at the real scale. Furthermore, through differentiated calibration of mechanical parameters, the interface weakness effect of the equivalent ITZ is consistent with the mechanical characteristics of the real ITZ. Upon completion of aggregate placement, the mesh property value of the aggregate regions is updated to 1, and ITZ with a thickness of 1 mm is generated along the outer edges of the aggregate grains, assigning its mesh property value to 2 (Figure 3b). Adjacent contacting aggregate grains may share the same ITZ region (Figure 5). Through iterative determination and updating the property value of the square background mesh, the grading and coordinate positioning of the three-phase medium for aggregate, mortar, and ITZ are completed. A two-dimensional random aggregate mesoscopic model for the concrete three-phase medium is ultimately constructed. The generation and accumulation process of the randomly irregular aggregate grains is shown in Figure 6.

### 2.3. Calibration of Mesoscopic Model Calculation Parameters

Figure 5 shows the schematic diagram of the uniaxial compression loading of concrete specimens. Previous mesoscopic simulation studies have indicated that the friction coefficient between the loading plate and the concrete cube specimen significantly affects the simulation results [33,34], with the compressive strength increasing as the upper friction coefficient increases. A reference point (RP) is established at the center node at the top and coupled with all nodes at the top. A quasi-static displacement of 0.6 mm is applied through the reference point to control the compressive loading. The bottom of the model is fixed, and the translational degrees of freedom in the *x* and *y* directions of all nodes are constrained. The left and right sides of the model are free boundaries without any constraints, allowing lateral free deformation induced by the Poisson effect. The calculation methods for stress and strain are consistent with the experiments: the average stress is obtained by dividing the bearing capacity of the reference point by the upper surface area of the specimen, and the average strain is determined by dividing the displacement of the reference point by the specimen’s compressive length. For quasi-static loading problems, the implicit algorithm accurately reflects the true stress state of the specimen without being affected by specimen inertia effects or loading kinetic energy. Numerical simulations are performed using the ABAQUS/Standard version 2022 implicit calculation module.

The Concrete Damage Plasticity (CDP) model built into ABAQUS assumes that the failure of concrete is jointly dominated by both tensile cracking and compressive crushing. This model introduces an isotropic damage variable to characterize stiffness degradation resulting from accumulated damage and the irreversible plastic deformation under external loads, which can effectively improve the simulation accuracy of the nonlinear mechanical response in concrete-like materials. In the numerical analysis of the meso-scale of concrete, the CDP model is often used to define the constitutive relationship of mortar and the ITZ [35,36]. This paper also uses this model to investigate the mesoscopic damage effects in concrete. The stress–strain relationships based on Cauchy stress and effective stress are expressed in Equations (5)–(7).

(5) $$\sigma =\frac{\left(1-d_{c}\right)D_{0}^{el}}{\left(\varepsilon -\varepsilon^{pl}\right)}=\frac{D^{el}}{\left(\varepsilon -\varepsilon^{pl}\right)}$$

(6) $$\bar{\sigma }=\frac{D_{0}^{el}}{\left(\varepsilon -\varepsilon^{pl}\right)}$$

(7) $$\sigma =\left(1-d_{c}\right)\bar{\sigma }$$

where *σ* and $\bar{\sigma }$ denote the stress and effective stress, respectively. *ε* and *ε*^pl^ represent the strain and plastic strain, respectively, $D_{0}^{el}$ indicates the initial stiffness and *d_c_* is the damage variable. The physical meaning of “*d*_c_” in this study is the effective elastic stiffness decay ratio caused by microcracks nucleation and propagation within the material. The damage variable presented in all figures and tables of this paper are quantified solely by the symbol “*d*_c_”.

The dominant failure mechanism of the ITZ is typically interfacial debonding, whereas the mortar mainly undergoes internal cracking. This study did not use the same CDP model settings for ITZ and mortar. Instead, it distinguished their different failure mechanisms through refined parameter differentiation. The elastic modulus and compressive/shear strength of ITZ are significantly reduced (lower than mortar). This allows it to initiate damage evolution under low stress, matching the low threshold and rapid development characteristics of interface peeling. Mortar employs higher strength and stiffness parameters, corresponding to its high internal cracking threshold and progressive failure characteristics. The CDP model can couple tensile, compressive, and shear damage, accurately characterizing the composite failure characterized by predominantly ITZ shear debonding and supplemented by localized tensile debonding. It can also reflect the initiation and propagation of microcracks within the mortar bulk phase. Both are quasi-brittle cumulative damage failures, and the constitutive framework of this model can achieve a unified characterization. This method has been validated by numerous classic studies on concrete microstructure simulation [2,35]. Furthermore, using a unified CDP model avoids artificial stress concentration at the junction of different constitutive models, ensuring the continuous transmission of the mechanical response of the three-phase system while also considering computational efficiency and adapting to the systematic analysis of multiple sets of stochastic models.

**Figure 7:**
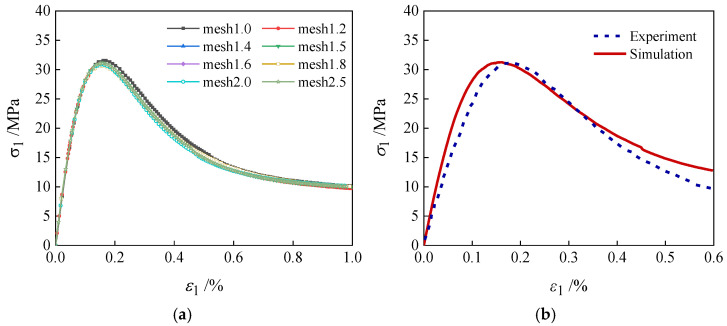
Uniaxial compression stress–strain curves from numerical simulations and experimental tests: (**a**) stress–strain; (**b**) comparison between simulation and experiment.

Upon the generation of the concrete geometric model, it is essential to select an appropriate mesh size to minimize the dependence of simulation results on mesh discretization. In this study, a mesh sensitivity analysis is conducted using eight different element lengths (*L*_e_) [37,38] with values of 1.0 mm, 1.2 mm, 1.4 mm, 1.5 mm, 1.6 mm, 1.8 mm, 2.0 mm, and 2.5 mm. Figure 7a presents the stress–strain curves of specimens with varying mesh sizes under uniaxial compression, with all calculation parameters set according to Table 1. To eliminate the influence of aggregate geometry, circular aggregate grains are employed in the simulations. The results indicate that the mesh size has a negligible effect on the development of initial stiffness in the macroscopic stress–strain curves, primarily influencing the peak stress and the post-peak softening behavior. However, the curves basically overlap, with no significant differences, indicating a weak model mesh dependence. At the peak stress level, the specimen with *L*_e_ = 1 mm exhibits slightly higher values, but the relative difference is less than 1%, falling within an acceptable error range. Considering the balance between computational efficiency and simulation accuracy, all the subsequent numerical simulations in this paper adopt a meshing scheme with an element length of 1 mm.

Through a combined approach of experimental testing [39] and parameter inversion, the mechanical parameters of coarse aggregate, fine aggregate, mortar, and ITZ are determined, thereby establishing a basis for subsequent meso-scale analysis. The experimental data in Figure 7, compared with the numerical simulation, are from the uniaxial compression test data for concrete conducted by Shang [39]. The concrete used in the experiment consisted of 368 kg/m^3^ of cement, 661 kg/m^3^ of river sand, 1176 kg/m^3^ of crushed stone, and 195 kg/m^3^ of water, with a water–cement ratio of 0.53. The ordinary Portland cement is PO42.5. The naturally formed medium sand with a fineness modulus of 2.8 is used. The coarse aggregate is crushed stone (particle size 10–20 mm). The cubic specimens are 100 × 100 × 100 mm in size. A vertical load is applied at a set loading rate of 10^−4^ s^−1^ until the specimen fails. The lateral pressure (tensile force), principal pressure (tensile force), and deformation of the specimen are dynamically acquired by a computer. The numerical simulation differs from the experiments in terms of the technical means, materials used, and loading methods. However, through sensitivity analysis, the mechanical properties of the numerical simulation can be made consistent with the experiments, thereby reflecting the validity of the numerical results.

The parameter inversion process is as follows: (a) Global search: Based on existing research results [40,41,42] on the microstructure parameters of concrete, the reasonable range of values for each parameter to be inverted is determined, and a set of initial parameters close to the optimal solution is obtained. (b) The obtained initial parameter set is used as the initial value of the quasi-Newton method, and local fine-grained iterative optimization is performed until the objective function converges to the minimum value, meeting the error evaluation index requirements. (c) Result verification: The optimized parameters are substituted into the microscopic model, and a uniaxial compression simulation is performed to verify the consistency between the simulation curve and the experimental curve. If the accuracy requirements are not met, the parameter range is re-adjusted, and the above process is repeated.

Figure 7b compares the numerical simulation results of concrete under uniaxial compression using a 1 mm mesh with experimental data. The simulated macroscopic stress–strain curve exhibits strong agreement with the experimental curve, particularly in the initial linear ascending stage and the hardening phase. The peak strengths are close, with an error within 10%, and the overall variation trend is consistent. A slight deviation is observed in the post-peak descending branch, which arises primarily from the use of a three-dimensional model in the experiment versus the two-dimensional model adopted in the present simulation. Such a discrepancy is deemed acceptable, and the overall consistency satisfies the requirements for analysis. In conclusion, the computational parameters for aggregate, mortar, and ITZ determined in this study are reliable and suitable for meso-scale numerical investigation into the mechanical behavior of concrete.

During the parameter calibration process, concrete aggregate is treated as a linear elastic material, with nonlinear behaviors and tensile or compressive damage excluded. The linear elastic assumption is physically justifiable, which substantially mitigates computational costs and enhances modeling efficiency. The linear elastic assumption of aggregate does not change the core mode of post-peak damage propagation in concrete, only having a slight impact on the damage propagation rate. The elastic modulus is set to 70 GPa, with a Poisson’s ratio of 0.23. For normal-strength grade concrete, the mortar compressive strength is taken as 35 MPa and the elastic modulus as 25 GPa. The macroscopic strength and stiffness of concrete are dominated by the mortar matrix. The ITZ, as a thin interface layer, has a limited direct contribution to the overall load-bearing capacity due to its strength variation. It only indirectly affects macroscopic mechanical behavior through damage initiation and propagation. Therefore, it is assumed that its strength has a relatively small direct impact on the overall concrete strength. This conclusion is consistent with the findings of the previous studies [4,5] on the mesoscopic concrete models. The strength of ITZ is determined using a strength reduction method. Prokopski and Halbiniak [43] conducted microscopic tests on cement-based materials and determined that the compressive strength of ITZ was 50% to 90% of that of mortar. This range has become a common basis for setting the ITZ strength in subsequent mesoscopic simulations of concrete [44,45,46,47]. This study ultimately selected 80% as the reduction factor, determined through parameter sensitivity analysis and experimental inversion calibration, ensuring a high degree of agreement between the simulation results and physical experiments. The mortar plastic damage variables are determined based on the recommended values of the ABAQUS concrete plastic damage model in the Abaqus Analysis User’s Manual and through experimental calibration. The plastic damage behaviors of the concrete matrix are characterized using the CDP model, the key parameters of which are listed in Table 1 [39]. The remaining parameters are assigned according to the recommended values in the ABAQUS help file [48]. The specific values are as follows: eccentricity 0.1, K coefficient 2/3, the ratio of uniaxial to biaxial compressive strength 1.16, and viscosity coefficient 1 × 10^−5^.

## 3. Results and Discussions

### 3.1. Effects of Aggregate Geometry and Random Distribution on the Mechanical Characteristics of Concrete

**Figure 8:**
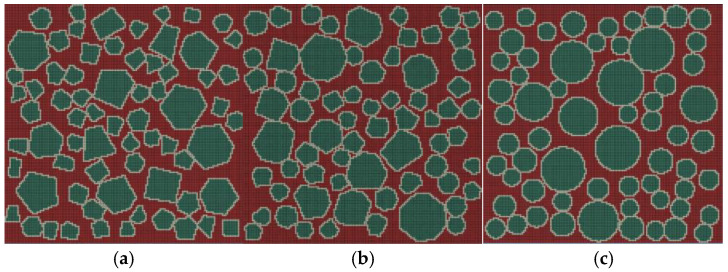
Schematic diagram of specimens with different aggregate geometries: (**a**) polygonal specimen 1-1; (**b**) polygonal specimen 2-1; (**c**) circular specimen 1.

Aggregate grains in concrete predominantly exist as polyhedra and ellipsoids and are widely used in actual reinforced concrete constructions [49]. To more accurately reflect the mesostructural characteristics of concrete, this study used three types of mesoscopic models for aggregate shapes: Polygon 1 (aggregate edges 4-6), Polygon 2 (aggregate edges 5-8), and circular aggregate. The three aggregate geometries are illustrated in Figure 8. The first digit of specimen numbering denotes the aggregate geometry type, while the second digit represents different random distribution conditions for aggregate grains of the same shape. Among them, specimen numbers 1-1, 1-2, and 1-3 represent randomly distributed polygonal aggregate grains with 4-6 sides, corresponding to sharp-edged aggregate grains (such as crushed stones). Specimen numbers 2-1, 2-2, and 2-3 represent randomly distributed polygonal aggregates with 5-8 sides, corresponding to rounded aggregate grains (such as pebbles). Specimen numbers 1, 2, and 3 represent randomly distributed spherical aggregate grains. The aggregate grains are generated in the order of larger particle size, followed by smaller particle size. The positions of aggregate grains within each size range are randomly generated, and the total area remains constant. Under the condition of maintaining the shape of the aggregate grains unchanged, the distribution position of each new aggregate grain is randomly generated each time. Therefore, samples with the same shape but different random distributions will be generated and are named 1, 2, and 3.

**Figure 9:**
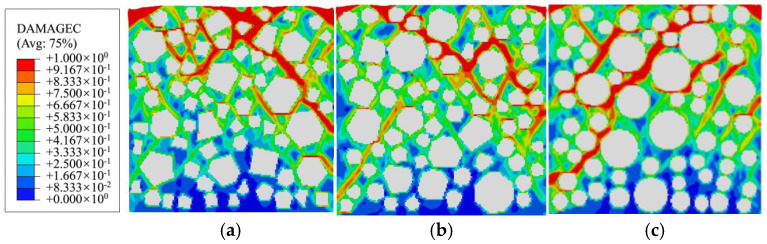
The compressive damage of specimens with different aggregate geometries: (**a**) polygonal specimen 1-1; (**b**) polygonal specimen 2-1; (**c**) circular specimen 1.

Figure 9 illustrates the final damage distribution characteristics of specimens with three different aggregate geometries under uniaxial compression. The damage variable presented in all figures and tables in this study is quantified solely by the symbol “*d*_c_”. A total of 50% damage (*d*_c_ = 0.5) represents a 50% loss of elastic stiffness for the meso-scale element, and the effective stiffness is only 50% of the initial value. The element is in a stage of continuous stiffness degradation, and the mechanical performance gradually decays (corresponding to the green area in the cloud chart). Additionally, 100% damage (*d*_c_ = 1.0) represents the complete loss of elastic stiffness for the meso-scale element, resulting in an effective elastic modulus of zero (*E*_d_ = 0). The element loses the ability to resist elastic deformation and is in a completely damaged state (corresponding to the red area in the cloud chart). The results demonstrate that aggregate geometry significantly influences the final damage distribution pattern in concrete. Moreover, the spatial distribution of coarse aggregate grains plays a regulatory role in the damage propagation state [33,50,51]. Upon specimen failure, a single coarsest or longest primary damage zone forms, and macroscopic failure primarily manifests as sliding or splitting along this primary damage zone. Specifically, the polygonal aggregate specimens exhibit a more tortuous and shorter primary damage zone. This is attributed to the angular structure of polygonal aggregate grains, which obstructs and redirects damage propagation paths. In contrast, specimens with circular aggregates display a more extensive primary damage band with greater penetration depth, owing to the absence of significant path obstruction effects.

The more severe asymmetry in the upper region of the damage distribution shown in the figure is caused by the coupling of the non-uniform stress transmission under top displacement loading, differences in lateral deformation constraints due to the Poisson effect, and microscopic stress disturbances caused by the random distribution of aggregate grains. The top displacement loading causes localized stress concentration during the downward stress transmission, leading to the initial initiation and accumulation of microcracks in the upper region. Furthermore, the weaker lateral deformation constraints in the upper region accelerate ITZ damage development. Due to the stress redistribution and lateral deformation constraints under fixed constraints, damage initiation is delayed, and development is slow in the lower region. The random distribution of aggregate grains provides the microstructural basis for this asymmetry, ultimately resulting in a significant difference in damage severity between the upper and lower parts.

**Figure 10:**
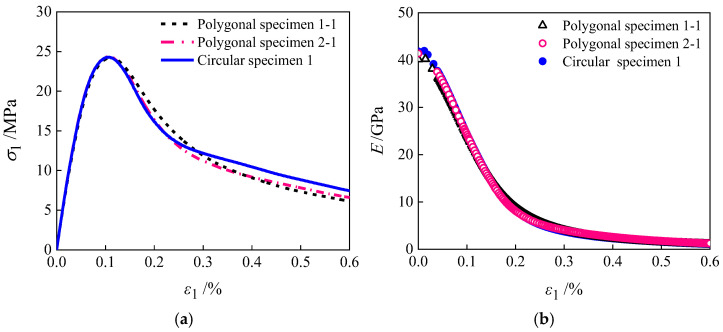
Stress–strain curves and modulus decay of specimens with different aggregate geometries: (**a**) stress–strain; (**b**) modulus decay.

Figure 10 presents the macroscopic stress–strain curves and the elastic modulus decay patterns of the specimens with three aggregate geometries. As shown in Figure 10a, the stress–strain curves of specimens exhibit essentially identical ascending segments and peak stresses, with relative errors in peak stress all below 1%. This indicates that the aggregate geometry has a negligible influence on both the elastic modulus and the peak strength of concrete [52], as these macroscopic mechanical properties are primarily determined by the strength characteristics of the mortar and the ITZ. However, the descending segments of the three curves exhibit distinct differences. This stems from the varying degrees of stress concentration induced by aggregate geometry within the ITZ. Such stress heterogeneity leads to an uneven internal stress distribution, thereby altering the initiation and propagation paths of damage. Figure 10b further reveals that the decay patterns of the elastic modulus of the three specimens are nearly identical, indicating that aggregate geometry has no significant effect on the modulus decay process of concrete.

During the preparation of concrete specimens, the geometry, size, and distribution of aggregate grains in the concrete exhibit random characteristics. While aggregate geometry and sizes can be precisely controlled by setting appropriate irregularity indices and scaling factors [53], the random distribution of aggregate grains remains difficult to regulate manually. This section further investigates the influence of random aggregate distribution on the mechanical properties and damage evolution patterns of concrete. To eliminate interference from variations in aggregate gradation, particle quantity, and size differences, the aforementioned parameters are strictly controlled to remain consistent throughout the experiments, with only the spatial arrangement of aggregate grains altered.

**Figure 11:**
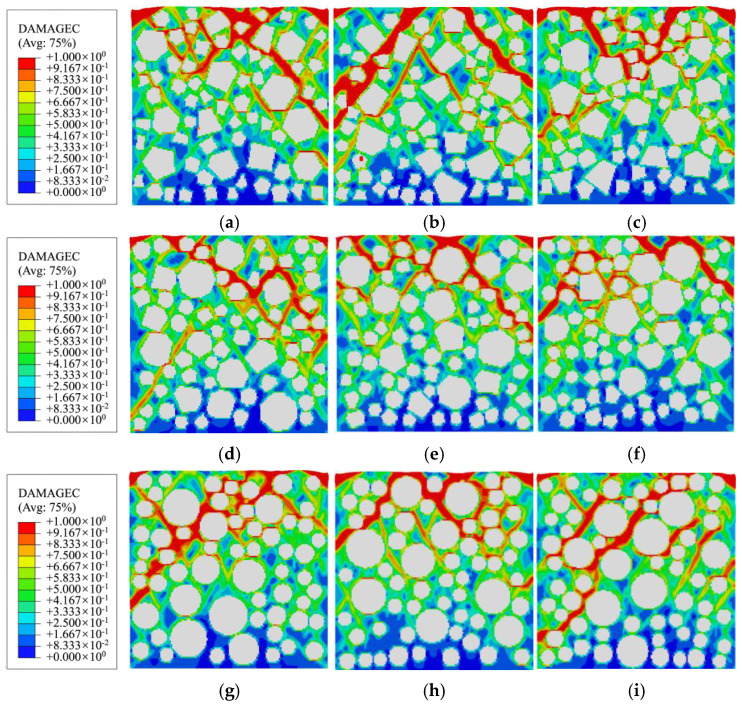
Compressive damage states of specimens with randomly distributed aggregate grains: (**a**) polygonal specimen 1-1; (**b**) polygonal specimen 1-2; (**c**) polygonal specimen 1-3; (**d**) polygonal specimen 2-1; (**e**) polygonal specimen 2-2; (**f**) polygonal specimen 2-3; (**g**) circular specimen 1; (**h**) circular specimen 2; (**i**) circular specimen 3.

Figure 11 presents the final damage distribution patterns of specimens with three aggregate geometries after uniaxial compression under random aggregate distribution conditions. The results indicate that the random distribution of aggregate grains significantly influences the final damage distribution morphology. All specimens exhibited typical diagonal shear failure patterns. Due to variations in the spatial distribution of aggregate grains, a number of initial damage zones develop, and their expansion directions differ, ultimately leading to variations in the orientation of the through cracks. Ultimately, all concrete specimens will form a main damage zone that is the thickest or longest. The macroscopic failure and destruction are mainly manifested as sliding or splitting along this main damage zone.

**Figure 12:**
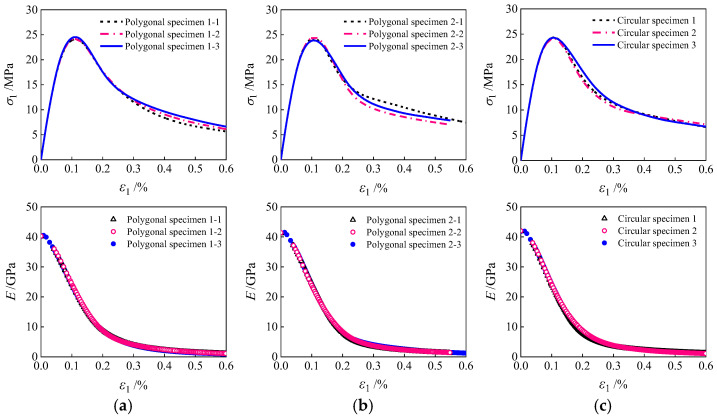
Stress–strain curves and modulus decay of specimens with randomly distributed aggregate grains: (**a**) polygonal specimens 1; (**b**) polygonal specimens 2; (**c**) circular specimens 3.

Figure 12 illustrates the macroscopic stress–strain curves and elastic modulus decay patterns of specimens with different aggregate geometries under random aggregate distribution conditions. As depicted, the random distribution of aggregate grains exerts a negligible influence on the elastic modulus decay process. The rising segments of stress–strain curves and peak stresses are essentially consistent across all specimens, with differences observed only in the falling segments. This indicates that the random arrangement of aggregate grains has minimal impact on both the elastic modulus and peak strength of concrete [54]. Further analysis, integrating the damage distribution characteristics shown in Figure 11, reveals that variations in the spatial distribution of aggregate grains primarily affect the initiation location and propagation direction of damage zones. Consequently, the final distribution of damaged regions within the specimens exhibits significant differences.

### 3.2. Mesoscopic Damage Evolution Law of Concrete

**Figure 13:**
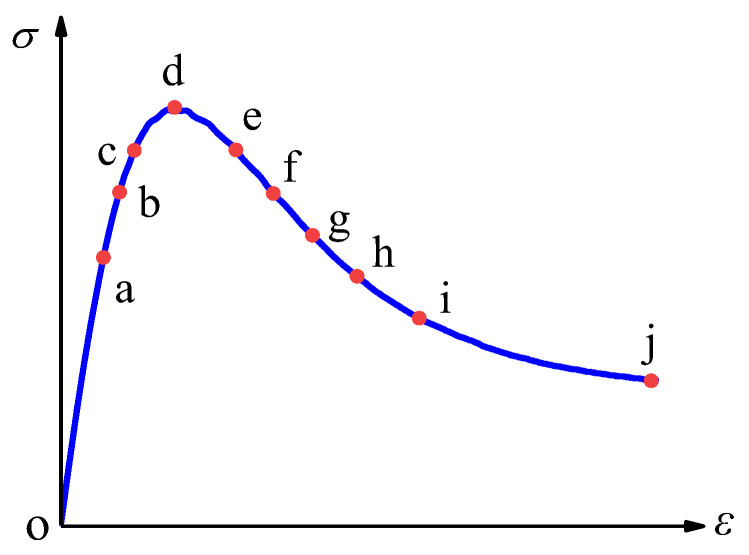
Marked points at different positions on the stress–strain curve.

Figure 13 annotates the characteristic points along the uniaxial compression stress–strain curve. Point *a* marks the endpoint of the linear elastic phase, while point *j* corresponds to the macroscopic failure point of the specimen at 1% axial strain. The intermediate points (*b*→*i*) are delineated based on the ratio of their respective stresses to the peak stress. The stress distribution at each marked point is detailed in Table 2.

**Figure 14:**
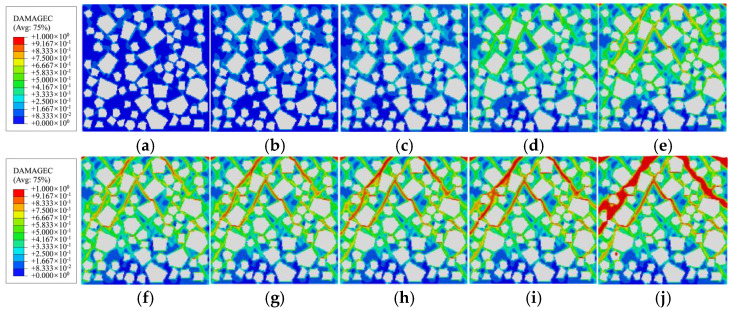
Damage evolution of polygonal aggregate specimen 1-2 during the compression process: (**a**) point *a*; (**b**) point *b*; (**c**) point *c*; (**d**) point *d*; (**e**) point *e*; (**f**) point *f*; (**g**) point *g*; (**h**) point *h*; (**i**) point *i*; (**j**) point *j*.

**Figure 15:**
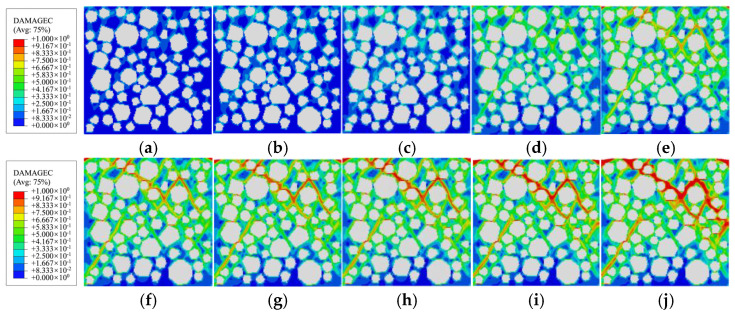
Damage evolution of polygonal aggregate specimen 2-1 during the compression process: (**a**) point *a*; (**b**) point *b*; (**c**) point *c*; (**d**) point *d*; (**e**) point *e*; (**f**) point *f*; (**g**) point *g*; (**h**) point *h*; (**i**) point *i*; (**j**) point *j*.

**Figure 16:**
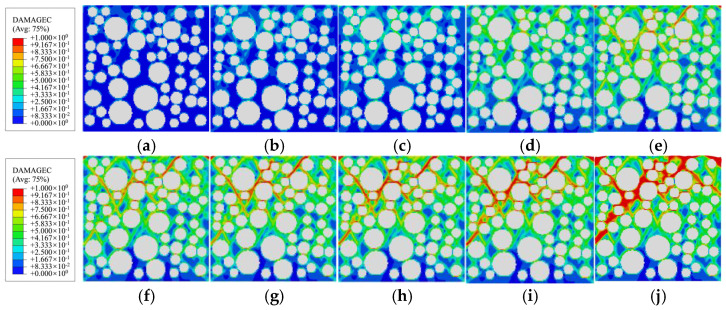
Damage evolution of circular aggregate specimen 1 during the compression process: (**a**) point *a*; (**b**) point *b*; (**c**) point *c*; (**d**) point *d*; (**e**) point *e*; (**f**) point *f*; (**g**) point *g*; (**h**) point *h*; (**i**) point *i*; (**j**) point *j*.

Figure 14, Figure 15 and Figure 16 respectively depict the complete process of crack initiation, propagation, and macroscopic failure under uniaxial compressive loading for polygonal specimens 1-2, 2-1, and circular aggregate grains 1 specimens. These illustrations precisely correspond to the meso-scale damage distribution at 10 characteristic points (*a*→*j*) along the stress–strain curve presented in Figure 13. In the legend, the red region indicates a damage variable *d*_c_ is approaching or equal to 1, indicating complete damage of the element and loss of load-bearing capacity [2]. The green region, where *d*_c_ is approximately 0.5, signifies the element undergoing stiffness degradation, with gradually diminishing mechanical properties. The blue region, with *d*_c_ ≈ 0.2, indicates negligible damage and quasi-intact mechanical behavior.

Combining the mesoscopic damage evolution contour plots in Figure 14, Figure 15 and Figure 16, the common patterns of concrete damage development and the specific characteristics induced by aggregate geometry can be clearly revealed. Damage progression occurs in three stages, all centered on the interface transition zone (ITZ). (1) Elastic stage (*o*→*a*): As a weak region, the ITZ initiates damage first, transitioning visually from an initial blue (*d*_c_ ≈ 0.2, with no significant damage) to a light green (*d*_c_ ≈ 0.5, with stiffness degradation) along the aggregate periphery. The phenomenon of damage initiation from the boundary is not caused by the boundary layer constraint, but rather by the slight stress concentration caused by the Poisson effect at the boundary under uniaxial compression, coupled with the ITZ weak zone. It has nothing to do with the boundary design method. (2) Plastic hardening stage (*a*→*d*): Damage extends from multiple ITZs and gradually connects, spreading from the ITZ into the surrounding mortar to form dispersed green damage patches. At the peak stress point d, damage remains predominantly confined to the ITZ, with interconnected ITZ damage exhibiting a network-like distribution. Meanwhile, mortar elements, due to their higher strength, show only stiffness degradation (green regions) and exhibit almost no red fully damaged elements (*d*_c_ ≈ 1). (3) Strain softening stage (*d*→*j*): Damage in the ITZ is almost fully developed (extensive red zones), then propagates rapidly into neighboring mortar elements. Scattered damage zones merge and intensify in color (light green→dark green→red), ultimately forming a continuous primary damage zone that leads to macroscopic structural failure.

**Figure 17:**
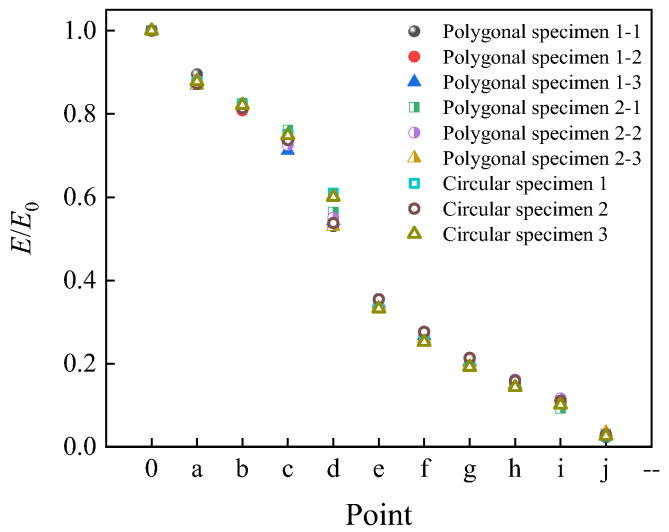
Ratio of modulus at marked point to initial modulus.

Figure 17 presents the ratio of stiffness at peak stress to initial stiffness for nine specimens covering all aggregate geometries and random distribution patterns. The figure indicates that the most significant stiffness decay occurs within the stress range of 90% peak stress (stage *c*→*d*→*e*). At the peak stress point d, stiffness values reach approximately 53–56% of the initial stiffness, indicating a 44–47% reduction in concrete stiffness. Consistent with previous analysis, point f marks the onset of complete damage failure. The maximum stiffness decay occurs prior to point f, where the elastic modulus at point f is only 26% of the initial value, signifying a 74% reduction in concrete stiffness.

### 3.3. Correlation Between Damage Energy and the Decay Characteristics of Strength and Stiffness

**Figure 18:**
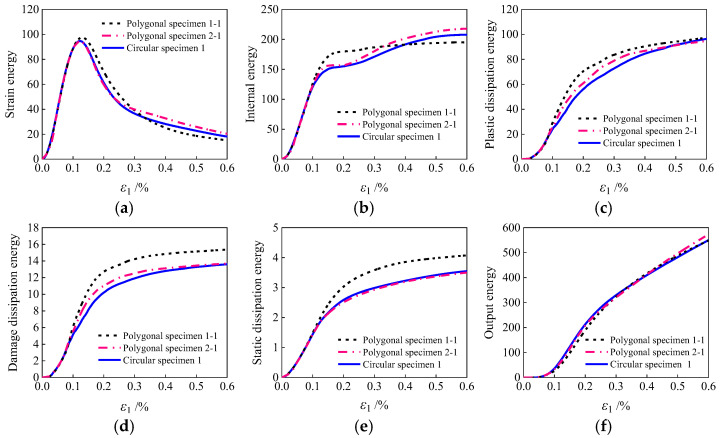
Energy change curves during the compression process of different aggregate geometries: (**a**) strain energy; (**b**) internal energy; (**c**) plastic dissipation energy; (**d**) damage dissipation energy; (**e**) static dissipation energy; (**f**) output energy.

As an inelastic material with damage energy dissipation, concrete exhibits a failure process that is fundamentally characterized by energy dissipation [55,56]. Figure 18 illustrates the variation patterns of six energy types with axial strain during the compression process for specimens with different aggregate geometries. The physical definitions and calculation methods for various types of energy are as follows. Strain energy is the energy stored in a concrete microstructure under external load during deformation. It is defined as: Ue=12∫σijVεijedV. Plastic dissipation energy is the energy consumed by irreversible plastic flow. It is calculated as: Up=∫σijVdεijpdV. Damage dissipation energy refers to the energy dissipated through the nucleation, propagation, and coalescence of microcracks within the material. It is defined as: Ud=∫12σijVεijedcdV. Internal energy denotes the total mechanical energy stored and dissipated by the microstructure, comprising the sum of strain energy, plastic dissipation energy, and damage dissipation energy. It is defined as: $U_{int}=U_{e}+U_{p}+U_{d}$. Static dissipation energy is the auxiliary energy dissipated in numerical simulations to account for material viscosity and contact interactions.

The results indicate that the sharpness of aggregate edges significantly regulates the energy evolution and damage progression within the mesoscopic model. During the elastic stage (axial strain 0–0.13%), the energy curves for specimens with various aggregate geometries essentially overlap, indicating a negligible influence of aggregate geometry. However, upon entering the strain softening stage, distinct differences in energy evolution emerge. The polygonal aggregate specimen 1-1 with sharp edges demonstrates the highest accumulation rate and final values in terms of plastic dissipation energy and damage dissipation energy, whereas the circular aggregate specimen exhibits the lowest values for all energy indicators. From a mesoscopic mechanism perspective, sharper aggregate edges induce more pronounced stress concentration, facilitating the initiation and propagation of cracks in the interfacial transition zone and mortar, thereby enhancing plastic and damage dissipation. In contrast, circular aggregate grains induce lower stress concentration and exhibit longer crack propagation paths, with energy primarily dissipated through plastic deformation and aggregate sliding, resulting in superior ductility. It should be noted that different aggregate geometries solely influence the energy distribution pathways, while the total dissipation energy ultimately converges, consistent with the principle of energy conservation.

It is noteworthy that the evolution of internal energy differs from other energy indicators. With increasing axial strain, the internal energy demonstrates an initial rise, followed by a decline and subsequent increase, undergoing an abrupt change at approximately 0.17% axial strain. This point corresponds to a stress level of about 80% of the peak stress (point *f* in Figure 13). This behavior indicates significant development of internal damage within the material, accompanied by a reduction in energy storage capacity. At point f, the elastic modulus is only 26% of its initial value. Coupled with the mesoscopic damage contour plots in Figure 14, Figure 15 and Figure 16, an evident fully damaged zone (marked in red) is observed. It is therefore inferred that when concrete strength decays to approximately 80% of its peak value, a fully damaged region forms within the specimen. Subsequent damage propagates continuously through this zone, ultimately forming a primary damage zone that leads to macroscopic failure.

**Figure 19:**
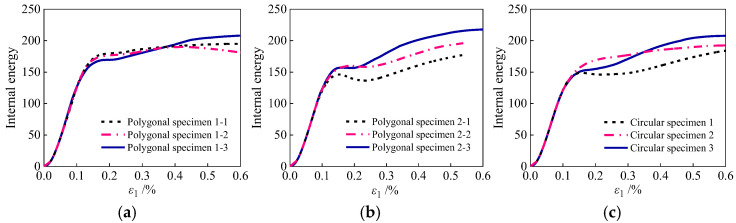
Curves of internal energy variation under random distribution of three shapes: (**a**) polygonal specimens 1; (**b**) polygonal specimens 2; (**c**) circular specimens.

Figure 19 shows the curves of internal energy changes under three random distributions of aggregate grains. As illustrated, the energy evolution curves for identical aggregate shapes under different random distributions consistently exhibit an inflection point, with minor variations in magnitude. This is attributed to the fact that variations in aggregate positioning dictate distinct damage propagation paths and distribution modes, thereby modulating the internal energy response. In conclusion, a critical inflection point exists in the internal energy evolution. A fully damaged zone develops within the specimen once the strain reaches the threshold range of 0.17% to 0.23%.

**Figure 20:**
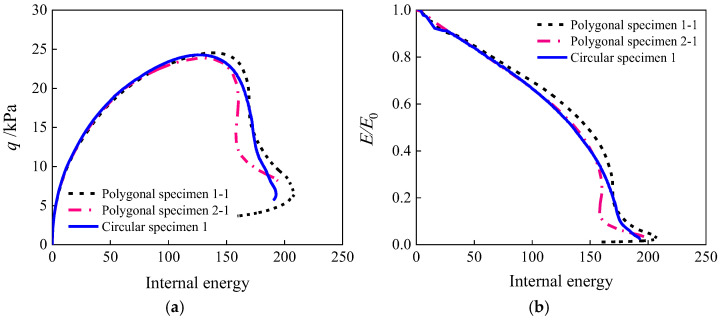
Correlation between internal energy and strength, stiffness: (**a**) stress-internal energy; (**b**) ratio-internal energy.

Figure 20 illustrates the stress-internal energy and modulus ratio-internal energy response patterns of concrete specimens with different aggregate geometries under uniaxial compression. As shown in Figure 20a, during the hardening stage (internal energy < 120), the specimen with circular aggregate grains exhibits a more gradual stress increase, a higher peak stress, and a greater amount of internal energy required to reach the peak. This reflects uniform contact and rational stress distribution of circular aggregate grains, which can delay energy dissipation. During the softening stage (internal energy > 120), the circular aggregate specimen demonstrates a more gradual stress decline and higher residual strength, whereas the polygonal aggregate specimen shows a steeper stress drop. This is attributed to the post-damage rolling and interlocking mechanism of circular particles that maintain load-bearing capacity, while the sharp edges of polygonal aggregate grains accelerate crack propagation. Figure 20b reveals that the modulus degradation curves nearly overlap during the initial elastic stage (internal energy <50), indicating that the initial stiffness is independent of aggregate geometry. Upon entering the damage evolution stage (internal energy >50), the modulus ratio of the polygonal aggregate specimen decays significantly faster than that of the circular aggregate specimen. This stems from stress concentration at the polygonal edges, which accelerates microcrack initiation and propagation. When the internal energy approaches 200, the modulus ratios converge to zero, signifying complete damage. The slightly earlier occurrence of full damage in polygonal aggregate specimens further confirms the more abrupt damage evolution in angular aggregate grains.

The fracture energy *G_f_* of concrete is defined as the energy dissipated per unit area of crack extension, serving as a fundamental parameter for characterizing its fracture resistance. In this study, the cumulative damage dissipation energy at the inflection point of internal energy is found to be equivalent to the critical fracture energy required for crack propagation under uniaxial compression (*G_f_* ≈ 120 N/m), indicating that this inflection point represents the critical energy state for the transition of microcracks into localized macrocracks. This conclusion aligns well with the fundamental criterion in fracture mechanics, which dictates that “crack propagation occurs only when the critical energy dissipation threshold is met”.

### 3.4. Quantitative Analysis of Concrete Damage Evolution

Upon entering the damage phase, microcracks initiate and progressively propagate within the concrete, resulting in a decay of the material’s elastic modulus as damage accumulates. The damaged elastic modulus at this stage is governed by the relation *E*_d_ = (1 − *d*_c_) × *E*_0_, where *E*_0_ denotes the initial undamaged elastic modulus. The damage elastic modulus *E*_d_ can be calculated from stress–strain data at various time steps, from which the damage variable *d*_c_ is derived, thereby enabling quantitative characterization of damage progression. This equation is the sole basis for calculating *d*_c_ in this article. The *d*_c_ of all specimens is derived using this equation, and there are no other calculation methods. The damage variable *d*_c_ of the concrete microstructure element characterizes the attenuation ratio of the elastic stiffness. When *d*_c_ = 0.9, the effective elastic modulus of the element is only 10% of its initial value, with stiffness nearly completely lost. And the stiffness is almost completely lost, unable to withstand further action of external loads. The microstructure element exhibits a complete failure state after the macroscopic crack penetrates, which conforms to the mechanical essence definition of “complete damage” in engineering. In the existing quantitative research on concrete microstructure damage [2,51], for the damage analysis of the CDP model, *d*_c_ = 0.85 and 0.95 are taken as the complete damage threshold. In this paper, the intermediate value *d*_c_ = 0.9 is selected, which is consistent with the threshold selection principle of the classic research in the field and has a reliable academic reference basis.

**Figure 21:**
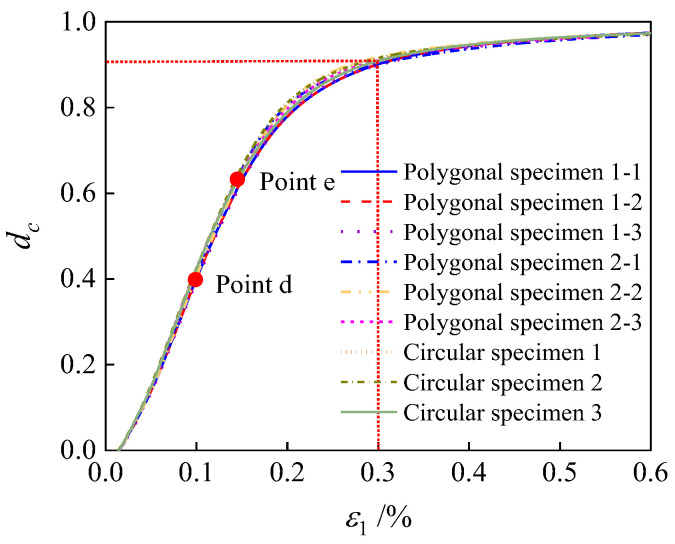
The variation curves of damage variables during the compression process of specimens with different aggregate geometries and random aggregate grain distributions.

Figure 21 illustrates the evolution curves of the damage variable for specimens with different aggregate geometries and random distributions. It can be observed that the fastest growth of the damage variable occurs between points d and e, corresponding to the stress drop from the peak value to 90% of the peak. This finding aligns with the accelerated stiffness decay observed in the same interval in Figure 17. Most specimens reach complete damage at *d*_c_ = 0.9, corresponding to an axial strain of approximately 0.3%, which corresponds to the *i*→*j* stage in Figure 13.

**Figure 22:**
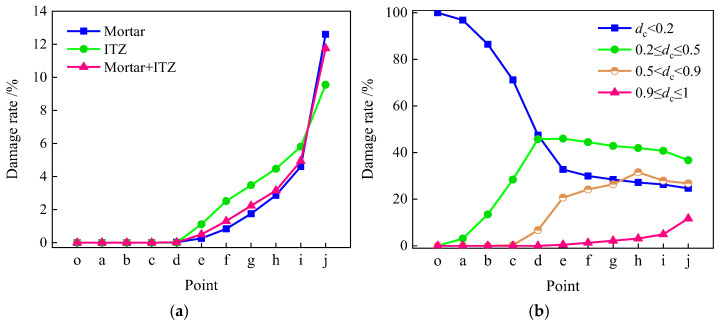
Curves of damage rate of each component and *d*_c_ in different ranges during the compression process for the circular aggregate specimen. (**a**) Damage rate of mortar, ITZ, and specimens; (**b**) damage rate of dc in different ranges.

The damage rate is defined as the ratio of the number of completely damaged units to the total number of units in the material. Under uniaxial compression, the damage rates of mortar, ITZ, and circular aggregate specimens are shown in Figure 22a. The figure indicates that ITZ elements sustain damage first, confirming that ITZ is the weak link in the compression damage of concrete. After the stress reaches the peak point d, the damage rates of the three curves increase significantly, and the growth rate accelerates, indicating that the damage enters the rapid accumulation stage. In the *d*→*i* stage, the damage rate of ITZ is always higher than that of the mortar matrix. After point *i* (stress drops to 50% of the peak stress), the damage rate of mortar exceeds that of ITZ. The overall damage rate of the specimen (mortar + ITZ) is highly consistent with the variation trend of ITZ, with values approximating the sum of both damage rates. This indicates that macro-damage in concrete results from the co-evolution of mortar and ITZ, with the damage evolution of the ITZ playing a dominant role in the overall damage progression.

Figure 22b shows that the damage rate of the circular aggregate specimen under uniaxial compression exhibits a staged and continuous evolutionary pattern. During the initial loading stage, damage is predominantly concentrated within the minor damage range (*d*_c_ < 0.2), primarily involving micro-crack initiation. During the mid-loading stage, the proportion of moderate damage range (0.2 ≤ *d*_c_ < 0.5) increased significantly, while the proportion in the minor damage range (*d*_c_ < 0.2) declined rapidly, indicating the transition from minor to moderate damage progression. During the final loading stage, the proportions of severe damage (0.5 ≤ *d*_c_ < 0.9) and complete damage (0.9 ≤ *d*_c_ ≤ 1) continue to increase. Micro-cracks coalesce to form macroscopic cracks, eventually leading to fully damaged elements. This progressive evolution from minor to complete damage reveals that damage in concrete undergoes a gradual damage process under uniaxial compression. Damage variables escalate continuously with increasing load, demonstrating the continuity and cumulative nature of damage evolution.

In conjunction with Figure 22a,b, it is evident that an intrinsic relationship exists between the mesoscopic interfacial damage and the macroscopic damage evolution in concrete: initial damage in the ITZ corresponds to the minor damage stage, while accelerated damage in the interface and matrix subsequently drives the progression toward severe and complete damage states. This confirms that damage accumulation in the ITZ is a critical factor driving the macroscopic damage development in concrete.

### 3.5. Influence of ITZ

The ITZ is a weak interface layer between the aggregate and mortar, with a thickness ranging approximately from 50 to 100 μm. Its mechanical properties typically fall below those of the adjacent mortar and aggregate. Due to the lack of experimental data on ITZ material properties and challenges in its implementation within finite element modeling, many meso-scale numerical models of concrete have omitted the inclusion of ITZ. To investigate the effects of ITZ on damage evolution, macroscopic stress–strain response, and modulus decay, this section compares two scenarios with and without ITZ.

**Figure 23:**
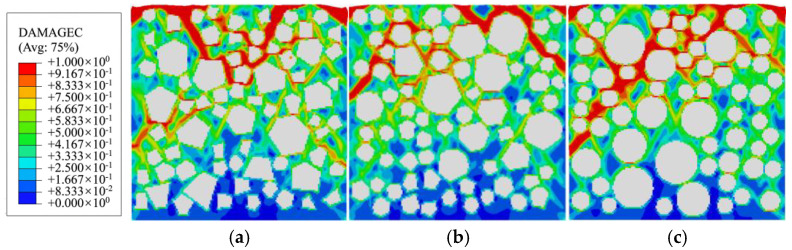

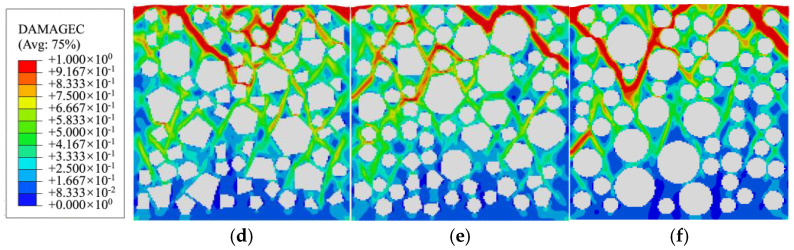
Damage distribution of three aggregate geometry specimens with or without ITZ: (**a**) polygonal specimen 1-3; (**b**) polygonal specimen 2-3; (**c**) circular specimen 1; (**d**) polygonal specimen 1-3 without ITZ; (**e**) polygonal specimen 2-3 without ITZ; (**f**) circular specimen 1 without ITZ.

Figure 23 compares the compressive damage distributions of specimens with three aggregate geometries under conditions with and without ITZ. The results indicate that, for polygonal aggregate grains, the presence or absence of ITZ has minimal effect on damage patterns. In both cases, damage propagates along similar directions and exhibits comparable characteristics, as aggregate edges remain the most vulnerable regions for stress concentration, regardless of ITZ consideration. In contrast, for circular aggregate grains, omitting ITZ makes mortar the weakest region, where damage propagation paths become more random, resulting in a damage pattern that markedly differs from that observed in models incorporating ITZ. Whether or not to consider ITZ (whether the aggregate grain intersects with the boundary), damage will first occur at the stress concentration location of the model, and its propagation path is entirely dominated by the internal aggregate characteristics, independent of the boundary layer. The final damage mode and damage evolution law are highly consistent, independent of the boundary layer. This boundary treatment method only has a slight impact on a small number of damage units at the local boundary and does not change the core laws of concrete microscopic damage evolution.

**Figure 24:**
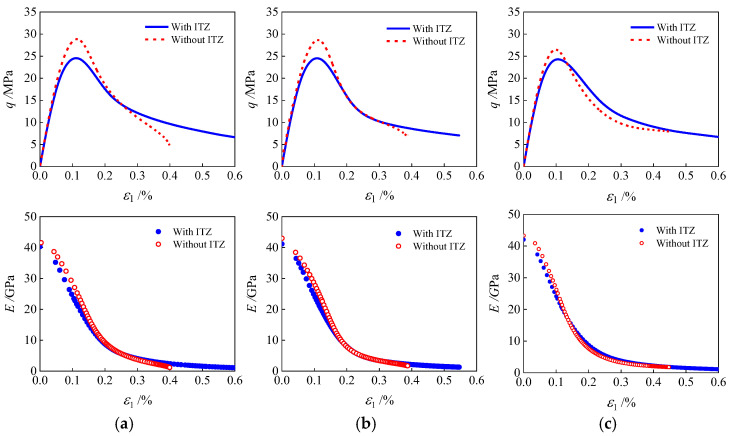
Stress–strain curves and modulus decay rules of three types of aggregate geometries specimens with or without considering the influence of ITZ: (**a**) polygonal specimen 1-3; (**b**) polygonal specimen 2-3; (**c**) circular specimen 1.

Figure 24 compares a comparative analysis of the corresponding stress–strain curves and modulus decay curves. (1) When the ITZ is not considered, the ascending stage (*o*→*d*) of the stress–strain curve becomes steeper, resulting in increased peak stress in concrete, enhanced overall stiffness, and slower decay. The peak strength of polygonal aggregate grains 1-3 and 2-3 and the circular aggregate grains 1 increased by 17.69%, 16.87%, and 9.12%, respectively, after ignoring the ITZ. The maximum increases in elastic modulus are 15.4%, 14.2%, and 9.8%, respectively. This behavior arises from the absence of the weak ITZ region, which inhibits micro-crack initiation and necessitates higher stress to induce failure and elevates peak stress. The extent of this increase in peak stress is influenced by aggregate morphology: the more irregular and angular the aggregate grains, the more pronounced the strength improvement, indicating that the effect of ITZ predominates over aggregate geometry. (2) In the softening stage (*d*→*j*), the stress decay rate of the ITZ-free model accelerates markedly, with this trend being more evident as aggregate edges become sharper. After ignoring the ITZ, the percentage of the peak stress drop slope (*E*) is greater than the percentage of the stress softening slope when ITZ is considered. That is, the drop slope after ignoring the ITZ is larger, indicating a faster strength attenuation. The ITZ can absorb the local stress through its own elastic deformation and early micro-damage, effectively reducing the local stress concentration coefficient at edges and preventing the rapid reaching of the mortar strength limit and the initiation of cracks. Without ITZ, the aggregate and the mortar directly contact each other, resulting in poor deformation coordination, and the stress at edges cannot be buffered through the interface, leading to an abrupt increase in the stress concentration effect. Once the stress reaches the mortar’s strength limit, cracks initiate rapidly from angular regions and propagate through the mortar instantaneously, resulting in explosive brittle failure. Consequently, sharper aggregate grains result in a steeper decline phase and more pronounced brittle characteristics in the non-ITZ model.

The ITZ constitutes an intrinsic weak region in concrete. The above analysis demonstrates that incorporating the ITZ in meso-scale numerical simulations is essential for accurately reflecting the nonlinear damage accumulation process and post-peak ductile behavior. Neglecting the ITZ may lead to a significant overestimation of concrete brittleness, potentially resulting in substantial misinterpretations in studies concerning fracture mechanics, dynamic response, and durability of concrete materials.

## 4. Conclusions

This study establishes a two-dimensional numerical model and analytical framework for concrete, accounting for randomly distributed convex aggregate particles and a three-phase structure comprising aggregate, mortar, and interfacial transition zone (ITZ). An efficient aggregate placement algorithm based on a background mesh is developed, enabling rapid model construction and precise subdivision. The influences of aggregate geometry, spatial distribution, and ITZ on macroscopic mechanical properties and damage evolution processes are systematically investigated. An associative mechanism between damage energy and the decay of both strength and stiffness is established. The damage rate and the variation characteristics of the damage variable *d*_c_ at each stage of mortar and ITZ are quantified. The key findings are summarized as follows:

1. An efficient and reliable meso-scale modeling framework for three-phase concrete has been established. Based on background meshes, the aggregate random placement algorithm achieves accurate screening and automated modeling of randomly convex polygonal aggregates, mortar, and ITZ. Mesh sensitivity analysis indicates low mesh dependency of the model, with a relative error in peak stress of less than 1%. Numerical simulations show good agreement with experimental stress–strain curves, with a peak stress error ≤10%.
2. The regulatory effects of aggregate shape and spatial distribution have been clearly elucidated. Aggregate geometry and spatial distribution exhibit minimal influence on the peak strength, elastic modulus, and modulus degradation process of concrete while predominantly governing post-peak mechanical behavior and damage patterns. Sharp edges of polygonal aggregate grains induce significant stress concentrations, where damage initiates preferentially within the corresponding ITZ. Rounded aggregate grains promote more uniform stress distribution, resulting in extended crack propagation paths and enhanced ductility.
3. Distinct characteristic points exist in the evolution of stiffness and damage variables. Within the stress range of 90% of the peak stress (the *c*→*d*→*e* stage), the stiffness decay of the specimen is most significant. At the peak stress point d, the stiffness has decreased by 44–47% (retaining 53–56% of the initial stiffness). The fastest damage variable growth coincided with the stress decline from peak stress to 90%, which coincides with the stage of maximum stiffness degradation. When the concrete strength decays to around 80% of the peak strength (axial strain ≈ 0.17%), a fully damaged zone appears inside the specimen, driving subsequent macroscopic failure. Most specimens attain complete damage at a damage variable *d*_c_ = 0.9 (axial strain ≈ 0.3%), corresponding to stresses below 50% of the peak stress.
4. The energy evolution and dissipation mechanisms are clearly elucidated. The sharper aggregate edges induce more pronounced stress concentration, facilitating the initiation and propagation of cracks, thereby enhancing plastic and damage dissipation. Rounded aggregate grains facilitate gradual dissipation through plastic deformation and particle sliding, exhibiting superior ductility. The evolution of internal energy differs from other energy metrics. Internal energy demonstrates an initial rise, followed by a decline and subsequent increase, with an abrupt transition occurring at approximately 0.17% axial strain (corresponding to 80% of peak stress), indicating a significant decline in the material’s energy storage capacity.
5. The ITZ is a key control mechanism in damage evolution. Damage first emerges within the ITZ, and its evolution dominates the macro-damage development of concrete. The overall damage rate of specimens highly correlates with the variations in ITZ and closely approximates their combined values. Neglecting the ITZ significantly overestimates the peak strength and stiffness of concrete, accelerates the decay rate of post-peak stress, and exacerbates brittle failure characteristics. These effects become more pronounced as the aggregate angularity increases.

The 2D model offers a clear and intuitive representation of the micro-pathways for crack initiation, propagation, and breakthrough. Its simplicity in modeling and computational efficiency make it well-suited for systematic analysis of micro-parameters and computational studies involving large sample sizes. The limitations are as follows: The 2D model cannot simulate the damage and failure mode of concrete under 3D complex stress conditions. It cannot reveal the spatial expansion characteristics of cracks in 3D space. The volume proportion of aggregate and the grading design of the 2D model have certain differences from the actual concrete structure. Based on the 2D modeling method presented in this study, a 3D meso-scale model of concrete will be constructed. The mesoscopic size effect of concrete will be explored by designing different-sized mesoscopic models of concrete, and the influence of aggregate particle size and sample size on the macroscopic mechanical properties and damage evolution laws will be systematically studied. The research on different loading paths and load types will be expanded. The model will be extended from quasi-static uniaxial compression to dynamic loads (such as impact, explosion), fatigue loads, freeze–thaw cycles, and other complex conditions.

## Figures and Tables

**Table 1:** Parameters of microscopic component materials.

Material	Young’s Modulus (GPa)	Poisson’s Ratio	Compressive Strength (MPa)	Shear Strength (MPa)
Aggregate	70	0.23	—	—
Mortar	25	0.20	35	3.5
ITZ	20	0.20	20	3.0

**Table 2:** The ratio of stress at the marked point to the peak stress.

Position	*a*	*b*	*c*	*d*	*e*	*f*	*g*	*h*	*i*	*j*
Ratio	linear end	80%	90%	100%	90%	80%	70%	60%	50%	damage

## Data Availability

The original contributions presented in the study are included in the article. Further inquiries can be directed to the corresponding author.
